# Evaluating competency-based medical education: a systematized review of current practices

**DOI:** 10.1186/s12909-024-05609-6

**Published:** 2024-06-03

**Authors:** Nouf Sulaiman Alharbi

**Affiliations:** 1 Department of Medical Education, College of Medicine, King Saud bin Abdulaziz University for Health Sciences, Riyadh, Saudi Arabia; 2 King Abdullah International Medical Research Center, Riyadh, Saudi Arabia; 3 Ministry of the National Guard - Health Affairs, Riyadh, Saudi Arabia

**Keywords:** Competency-based medical education, Program evaluation, Undergraduate medical education, Postgraduate medical education, Curriculum development

## Abstract

**Background:**

Few published articles provide a comprehensive overview of the available evidence on the topic of evaluating competency-based medical education (CBME) curricula. The purpose of this review is therefore to synthesize the available evidence on the evaluation practices for competency-based curricula employed in schools and programs for undergraduate and postgraduate health professionals.

**Method:**

This systematized review was conducted following the systematic reviews approach with minor modifications to synthesize the findings of published studies that examined the evaluation of CBME undergraduate and postgraduate programs for health professionals.

**Results:**

Thirty-eight articles met the inclusion criteria and reported evaluation practices in CBME curricula from various countries and regions worldwide, such as Canada, China, Turkey, and West Africa. 57% of the evaluated programs were at the postgraduate level, and 71% were in the field of medicine. The results revealed variation in reporting evaluation practices, with numerous studies failing to clarify evaluations’ objectives, approaches, tools, and standards as well as how evaluations were reported and communicated. It was noted that questionnaires were the primary tool employed for evaluating programs, often combined with interviews or focus groups. Furthermore, the utilized evaluation standards considered the well-known competencies framework, specialized association guidelines, and accreditation criteria.

**Conclusion:**

This review calls attention to the importance of ensuring that reports of evaluation experiences include certain essential elements of evaluation to better inform theory and practice.

**Supplementary Information:**

The online version contains supplementary material available at 10.1186/s12909-024-05609-6.

## Background

Medical education worldwide is embracing the move toward outcome-based education (OBME) [1, 2]. One of the most popular outcome-based approaches being widely adopted by medical schools worldwide is competency-based medical education (CBME) [3]. CBME considers competencies as the ultimate outcomes that should guide curriculum development at all steps or stages—that is, implementation, assessment, and evaluation [3–5]. To embrace CBME and prepare medical students for practice, medical educators usually utilize an organized national or international competency framework that describes the abilities that physicians must possess to meet the needs of patients and society. There are numerous global competency frameworks that reflect the characteristics of a competent doctor, for example, CanMEDS, Scottish Doctor, Medical School Projects, ACGME Outcome Project, the Netherlands National Framework, and Saudi Meds [1, 6–8].

With the worldwide implementation of CBME and availability of different competency frameworks, educators are expected to evaluate various modifications made to existing medical curricula [9, 10]. Such evaluation is intended to explore whether the program is operating as planned and its outcomes are achieved as intended in comparison to predetermined standards as well as to ensure improvement [11–13]. Furthermore, program evaluation revolves around two main concepts, that is, merit and worth [12, 14]. In 1981, Guba and Lincoln explained that the merits of a program are intrinsic, implicit, and independent and do not refer to a specific context or application, while evaluating a program’s worth entails judging the value of any aspect of it in reference to a certain context or precise application [12, 14].

To enable educators to determine the merits and worth of an educational program or curriculum, evaluation experts have proposed several models [14, 15]. Evaluation models are guiding frameworks that demonstrate what appropriate evaluation looks like and detail how it should be designed and implemented [16]. Although almost all evaluation models focus on exploring whether a program attains its objectives, they vary in numerous aspects, including their evaluation philosophy, approaches, and the specific areas that they encompass [17].

It is essential that educators choose a suitable evaluation model when they implement CBME, as the right model will enable them to pinpoint [15, 18, 19]. In other words, a program helps identify areas of success, challenges, and opportunities for improvement in CBME implementation, leading to a deeper comprehension of CBME strategies and their effectiveness. Moreover, implementing CBME demands significant efforts and a wide range of financial, human, time, and infrastructure resources [20]. Thus, ensuring that these efforts and resources are well utilized to enhance educational and healthcare outcomes is crucial. In addition, evaluation provides valuable evidence for accreditation, quality assurance, policies, and guidelines. Otherwise put, it supports informed decision-making on many levels [21]. On another front, sharing evaluation results and being transparent about evaluation processes can enhance public trust in available programs, colleges, and universities [19]. However, deciding which evaluation model to adopt can be challenging [9].

Not only can it be difficult to select an appropriate model to evaluate a CBME program, but CBME evaluation itself has numerous challenges, particularly given the lack of a common definition or standardized description of what constitutes a CBME program [9, 22, 23]. The complexity of CBME further tangles evaluation efforts, given the multilayered nature of CBME’s activities and outcomes and the need to engage a wide variety of stakeholders [11]. Moreover, the scarcity and variable quality of reporting in studies focusing on the evaluation of CBME curricula exacerbate these challenges [24]. Furthermore, few published articles provide a comprehensive overview of available evidence on the topic.

This review is therefore designed to synthesize the findings of published studies that have reported CBME evaluation practices in undergraduate and postgraduate medical schools and programs. Its objective is to explore which CBME program evaluation practices have been reported in the literature by inspecting which evaluation objectives, models, tools, and standards were described in the included studies. In addition, the review inspects the results of evaluations and how they were shared. Thus, the review will serve in supporting educators to make evidence-based decisions when designing a CBME program. In addition, it will provide a useful resource for educators to embrace what was done right, learn from what was done wrong, improve many current evaluation practices, and compare different CBME interventions across various contexts.

## Methods

Following a preliminary search within relevant journals for publications addressing evaluation practices utilized to assess competency-based curricula in medical education, the researcher used the PEO (participant, educational aspect, and outcomes) model to set and formulate the search question [24] as follows: *participants*: healthcare professionals and healthcare profession students; *educational aspect*: CBME curricula; *outcome*: program evaluation practices.

Next, the researcher created a clear plan for the review protocol. This review is classified as a systematized review rather than a systematic review [25]. While it does not meet the criteria for a systematic review because it relies on a single researcher and does not evaluate the quality of the studies included, it adheres to most of the steps outlined in the “Systematic Reviews in Medical Education: A Practical Approach: AMEE guide 94” [26]. Moreover, the researcher met with a medical educator with a strong background in CBME, an expert in review methods, and a librarian who is an expert in available databases and provided guidance and support for navigating such databases. Feedback was obtained from all three and used to finalize the review protocol. The protocol was followed to ensure that the research progressed in a consistent and systematized manner.

For this review, full-text articles published in peer-reviewed journals in English from 1 January 2000 to 31 December 2022 were searched within the following electronic data bases: PubMed, ERIC, Education Source, and CINHAL. The following terms were utilized to conduct the search: (Competency Based Medical Education OR Outcome Based Medical Education) AND (Evaluation OR assessment) AND (Undergraduate OR Postgraduate) AND (Implementing OR Performance OR Framework OR Program* OR Project OR Curriculum OR Outcome) (Additional file 1).

The researcher included articles that were published in English and reported evaluation practices for CBME or OBME curricula whether for undergraduate or postgraduate healthcare professionals. The researcher did not consider research reviews, commentaries, perspective articles, conference proceedings, and graduate theses in this review. In addition, articles that addressed students’ assessments rather than program evaluation were not included. Furthermore, articles that focused on teaching a particular skill (e.g., communication skills) or specific educational strategies (e.g., the effectiveness of Problem Based Learning) were excluded from this review.

To facilitate the screening of articles and ensure the process was properly documented, an online review software that streamlines the production of reviews (Covidence) was utilized, and all the lists of articles retrieved from the specified databases were uploaded to the tool website (available at www.covidence.org). The tool set the screening to start with the titles and abstracts then to proceed to full texts. During these stages, the reasons for excluding an article were precisely noted. Moreover, the PRISMA diagram (available at http://www.prisma-statement.org/) was produced by Covidence to illustrate the process of screening and including articles in this review.

After the decision was made to include an article, a data extraction tool created for the purpose of this review was used (Additional file 2). Since the term “program evaluation practices” is general and does not clearly define the method or focus of the analysis involved in critiquing evaluation efforts, the analysis of available evaluation practices in this review was based on the Embedded Evaluation Model (EEM) provided by Giancola (2020) for educators to consider when embedding evaluations into educational program designs and development [27]. The EEM outlines several steps. In the first step, “Define,” educators are expected to build an understanding of the evaluated program, including its logic and context. In the second step, “Plan,” educators must establish the evaluation-specific objectives and questions and select the model or approach along with the methods or tools that will be utilized to achieve those objectives. The next step, “Implement and Analyze,” requires educators to determine how the data will be collected, analyzed, and managed. In the fourth step, “Interpret the Results,” educators are expected to derive insights from the results in terms of how the evaluation can help with resolving issues and improving the program as well as how the results should be communicated and employed. Finally, in the “Inform and Refine” step, educators should focus on applying the results to realize improvements to the program and promote accountability [27].

In addition to supporting the aim of the current review, the theoretical insights from Giancola (2020) help to ensure alignment with best practices in curriculum evaluation. Thus, for each article, the extraction tool collected the following information: the author, the publication year, the country and name of the institution that implemented the CBME curriculum, the aim and method of the article, the type of curriculum based on the health profession specialty (e.g., medicine, nursing), the level of the curriculum (postgraduate or undergraduate), the evaluation objective, the approach/model or tool, the evaluation standard, the evaluation results, and the sharing of the evaluation results. The extracted information points are essential to contextualize the evaluation and allow educators to make sense of it and adapt or adjust it to their own situations. Understanding the context of an evaluation is important considering the wide variety of available educational environments, the diversity of evaluators, and the differences in goals, modes, and benchmarks for evaluation, all of which influence how an evaluation is framed and conducted [27].

The ***author, publication year***, and name and country of the ***institution*** that implemented the CBME curriculum provide identifiers for the original article and enable educators to seek further information about a study. The ***aim and method of the article*** were highlighted because they clarify the general context in which the evaluation was conducted. For example, this information can help educators understand whether an evaluation was carried out as a single action in response to a certain problem or was a phase or part of a larger project. The ***type of curriculum*** based on the health profession specialty (e.g., medicine, nursing) along with the ***level of the curriculum*** (postgraduate or undergraduate) have specific implications related to the nature of each specialty and the level of the competencies associated with the advancement of the program. All of the previously mentioned information is vital for educators to define and understand the program they are aiming to evaluate, which is the first step in the EEM. The ***evaluation objective***, ***approach/model or tool***, ***evaluation standard***, ***evaluation results***, and ***sharing of the evaluation results*** help to answer the research question of the current review by dissecting various aspects of the evaluation activities. In addition, the reporting of these aspects provides valuable insight into evaluation directives, plans, and execution. For educators, the ***evaluation objective*** usually clarifies the focus of the evaluation (e.g., how the program was implemented, the action done to execute education or outcomes of the program, and its effectiveness). The ***approach/model or tool*** of an evaluation is a core element of the design and implementation of the evaluation, as it determines the theoretical guidelines that underlie the evaluation and the practical steps for its execution. Based on the evaluation standard, which refers to the target used to compare the evidence or results of the evaluation, educators can judge the relevance of the evaluation to their own practices or activities. This information aligns with steps two and three of the EEM. The ***evaluation results*** are the results of the evaluation, which form the cornerstone for emerging solutions or future improvements. Finally, ***sharing the evaluation results***, or communicating the evaluation, is a key part of handling the results and working toward their application. This information is aligned with steps four and five of the EEM.

## Results

### Search results

**Fig. 1:**
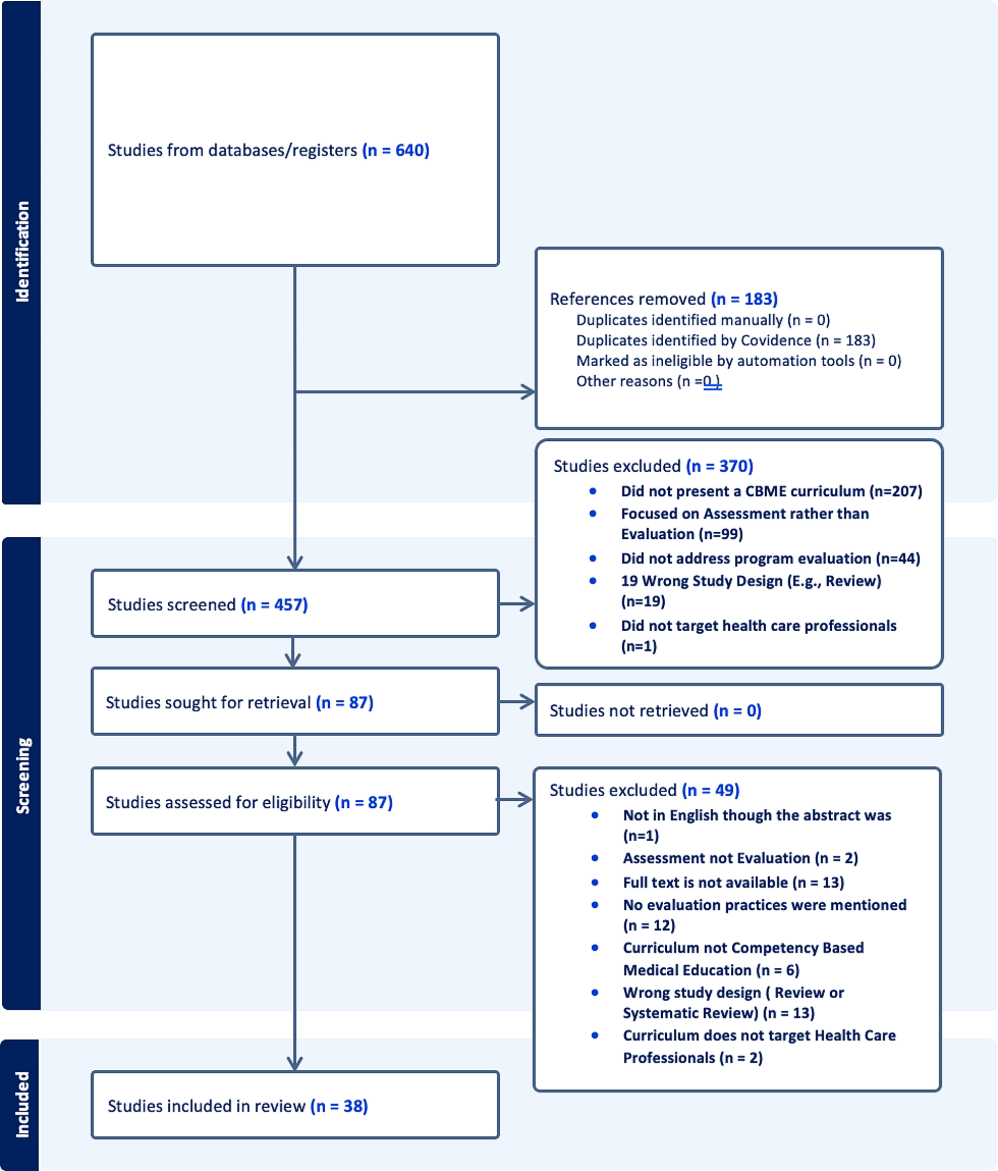
Flowchart illustrating the process of including articles in the review

Searching the identified databases revealed a total of 640 articles, and 183 total duplicates were removed. A total of 457 articles was considered for screening (371 PubMed, 13 ERIC, 23 Education Source, 50 CINHAL) (Fig. 1). Of those articles, 87 were retrieved for full-text screening. Ultimately, 38 studies met the inclusion criteria and were considered eligible to be included in the current review.

### Findings of the included studies

The 38 studies that met the inclusion criteria were published between 2010 and 2021, and the majority (15%; *n* = 6) were published in 2019. The studies represented the following countries: Canada (37%, *n* = 14) [10, 11, 28–39], USA (27.5%, *n* = 11) [40–50], Australia (5%, *n* = 2) [51, 52], China (5%, *n* = 2) [53, 54], Dutch Caribbean islands (2.5%, *n* = 1) [55], Germany (2.5%, *n* = 1) [56], Guatemala (2.5%, *n* = 1) [57], Korea (2.5%, *n* = 1) [58], the Netherlands (2.5%, *n* = 1) [59], New Zealand (2.5%, *n* = 1) [60], The Republic of Haiti (2.5%, *n* = 1) [61], Turkey (2.5%, *n* = 1) [62], and the region of West Africa (2.5%, *n* = 1) [63].

**Fig. 2:**
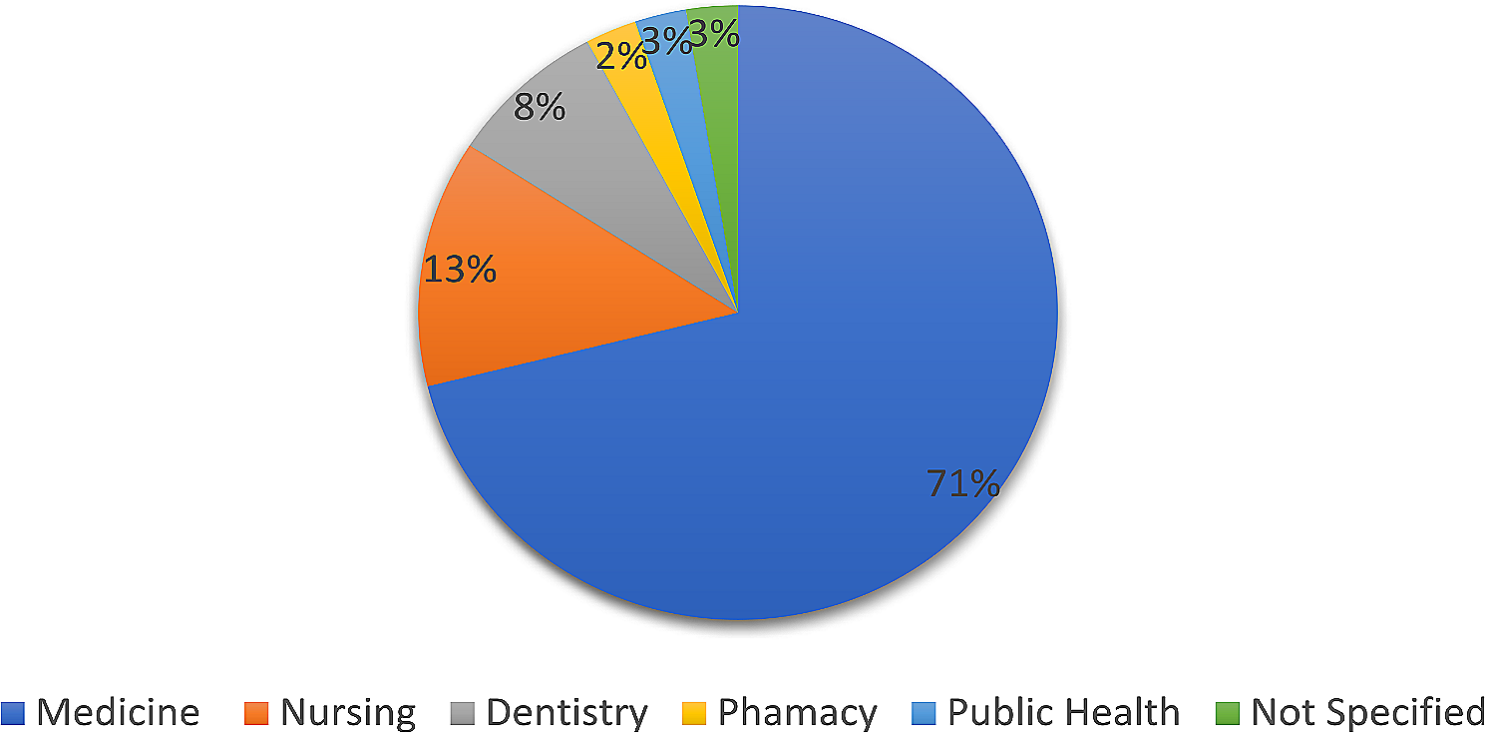
Curricula specialties in included articles

According to the evidence synthesized from the included studies, most of the evaluation practices were reported in competency-based curricula that targeted the level of postgraduate professionals (57%, *n* = 22) and were medical in nature (71%, *n* = 27) (Fig. 2).

The findings showed that 37% (*n* = 14) of the articles did not report the precise objective of evaluating the curriculum. Moreover, 84% (*n* = 32) did not report the evaluation approach or model used to assess the described curricula. The approaches or models reported include Pawson’s model of realist program evaluation [37], theory-based evaluation approaches [10], Stufflebeam’s context, inputs, processes, and products (CIPP) model [62], the concerns-based adoption model, sensemaking and outcome harvesting [33]the CIPP model [48], and quality improvement (QI) for program and process improvement [50]. On the other hand, a wide variety of evaluation tools was reported including observations (3%, *n* = 1) [28] surveys or questionnaires (58%, *n* = 22) [10, 28, 29, 31, 34–36, 38, 39, 41, 42, 45, 49–53, 55, 56, 58, 59, 63] interviews (16%, *n* = 6) [10, 28, 37, 41, 47, 62], focus groups (13%, *n* = 5) [35, 37, 41, 50, 59], historical document review or analysis (8%, *n* = 3) [10, 29, 33], educational activity assessment or analysis of the activity by separate reviewers (5%, *n* = 2) [55, 61], stakeholder discussions or reports about their inputs (5%, *n* = 2) [43, 44], curriculum mapping (3%, *n* = 1) [32], feedback from external reviews from accrediting bodies (3%, *n* = 1) [32], the Dundee Ready Education Environment Measure (DREEM) (3%, *n* = 1) [56], and students’ or participants’ assessments (5%, *n* = 2) [38, 46].

Of the studies, 37% (*n* = 14) utilized multi methods [10, 28–30, 32, 34, 35, 37, 38, 41, 48, 52, 56, 59]. Furthermore, 7.8% (*n* = 3) of the studies reported the nature of the tool, for example, quantitative or qualitative, without specifying the exact tool utilized [57, 60]. Moreover, 63% (*n* = 24) of the studies included in this review did not report the evaluation standards applied while assessing the competency-based curricula addressed. Yet, those studies that reported their standards were stated in various ways as follows: some publications referred to the standards of specific specialized associations or societies, such as the American Academy of Family Physicians and College of Family Physicians Canada [61], Royal Australian and New Zealand College of Psychiatrists [60], and The American Association of Occupational Health Nurses [45]. Other publications utilized known competency frameworks as their standards, such as CanMED [36, 37, 59], or the competencies of the American Board of Surgery [43] Association of Canadian Faculties of Dentistry [31], Royal College of Ophthalmologists [52], The Florida Consortium for Geriatric Medical Education [50], or the Dutch Advisory Board for Postgraduate Curriculum Development for Medical Specialists [59]. Furthermore, many of the publications referred to accreditation standards, such as the Accreditation Standards of the Australian Medical Council [51], Competencies of Accreditation Council for Graduate Medical Education [43], Accreditation Body in the Competency-based Curriculum [32], and the Commission on Dental Accreditation of Canada [31]. All the publications included in the review reported the results of their evaluations.

Finally, the results revealed that almost half (52.6%, *n* = 20) of the authors of the articles mentioned that they were publishing their experience with the intent of sharing lessons learned, yet they did not refer to any other means of sharing the results of their evaluations. In contrast, the other half did not mention any measures taken to communicate and share the evaluation results. Additional file 3 includes the characteristics and details of the data extracted from the studies included addressing evaluation practices in healthcare professionals’ education.

## Discussion

Evaluating a curriculum appropriately is important to ensure that the program is operating as intended [13]. The present study aimed to review the available literature on the evaluation practices of competency-based undergraduate and postgraduate health professionals’ schools and programs. This review inspected which evaluation objectives, models, tools, and standards were described as well as the results of evaluations and how the results were shared. The synthesized evidence indicates that most of the programs reporting evaluation practices were postgraduate-level medical programs. This focus on CMBE among postgraduate programs can be related to the fact that competency-based education is organized around the most critical competencies useful for health professionals after graduation. Thus, they are better judged at practice [64–66]. Moreover, although competency-based curricula were introduced to many health professions over 60 years ago, such as pharmacology and chiropractic therapy, within the medical field they have only evolved in the last decade [67].

Furthermore, the data revealed that there is a discrepancy in how evaluation practices were reported in the literature in terms of evaluation objectives, approaches/models, tools, standards, documenting of results, and communication plans. Each area will be further discussed in the following paragraphs considering the ten-task approach and embedded evaluation model [27, 68]. Both guide evaluation as an important step in curriculum development in medical education, detail the evaluation process, and outline many important considerations from design to execution [27, 68].

Evaluation is a crucial part of curriculum development, and it can serve many purposes, such as ensuring attaining educational objectives, identifying areas of improvement, improving decision-making, and assuring quality [13, 27]. Consequently, when addressing evaluation, it is important for educators to start by explaining the logic of the curriculum by asking, for example, what the program’s outcomes are and whether it is designed for postgraduates or undergraduates [27]. Moreover, educators must be precise in setting evaluation objectives, which entails answering certain questions: who will use the evaluation data; how will the data be used at both the individual and program level; will the evaluation be summative or formative; and what evaluation questions must be answered [27, 68, 69] However, many of the studies included in this review did not clearly explain the context of the curricula or report the objectives of their evaluation endeavors; rather, they settled for clarifying the objectives of the study or of the publication itself. One reason for this is that evaluation and educational research have many similarities [13] Nevertheless, the distinction between the two should be clarified, as doing so will enable other medical educators to better understand and benefit from the evaluation experience shared. Moreover, since CBME outcomes are complicated and should be considered on many levels, evaluation plans should include a focus, level, and timeline. The focus of an evaluation can be educational, with outcomes relevant to learners, or clinical, with health outcomes relevant to patients. The level of an evaluation can be micro, meso, or macro, targeting an individual, a program, or a system, respectively. The timeline of an evaluation can investigate outcomes during the program, after the program (i.e., how well learners have put what they learned in a CBME program into practice), and in the long term (i.e., how well learners are doing as practicing physicians) [70].

Once the evaluation objectives are clearly identified and prioritized, it is logical to start considering the evaluation approach or model that is most appropriate to attain these objectives considering the available resources. In other words, evaluation design should be outlined [27, 68]. The choice of an evaluation approach or model affects the accuracy of assessing certain tasks carried out by or to specific subjects in a particular setting [68, 71–75] This accuracy is referred to as an evaluation’s internal validity. Yet, the external validity of an evaluation entails that the evaluation results are generalizable to other subjects and other settings [68]. Each model has its own strengths and weaknesses, which require careful examination when planning an evaluation [14, 73–75]. Explaining and justifying why a particular evaluation approach was chosen for a specific curriculum can enrich the lessons learned from the evaluation and aid other educators. Furthermore, some of the available models were more utilized within various educational contexts than others [17] that calls for a continuous documentation of the evaluation approaches or models used to inform theory and practice. Considering the importance of reporting the approaches and models used, it is unfortunate that most of the publications did not indicate the approach/model they used for evaluation, which limits educators’ abilities to utilize the plans and build on their evidence.

Another critical task in the evaluation process is deciding on the measurement tool or instrument to be used. The tool choice will determine what data will be gathered and how they will be collected and analyzed [27, 68]. Thus, the choice should consider the evaluation objective as well as the uses, strengths, and limitations of each tool. The evidence in this review indicates that questionnaires or surveys were the most utilized tools in evaluating competency-based curricula. This result can be attributed to the advantages of this method (for example, it is a convenient and economical tool that is easy to administer and analyze and can be utilized with many individuals) [27, 68]. Nevertheless, it is important to highlight that questionnaires and surveys usually target attitudes and perceptions, which usually entails only a surface-level evaluation, according to the Kirkpatrick model [76]. The results also showed that in around 50% of the mixed-methods evaluations, the questionnaires were combined with another tool, such as interviews or focus groups. Understandably, utilizing an additional tool aims to deepen the level of the evaluation focus to include learning, behaviors, or results [76].

The evaluation evidence must be compared with a standard or target for educators to judge the program and make decisions [12]. Standards can be implicit or explicit, but they usually provide an understanding of what is ideal [12]. Worryingly, the results of this review revealed that many of the included studies did not clarify the standards they used to judge different CBME curricula. However, the studies that reported their standards used accreditation criteria or broad competencies frameworks, such as CanMeds, which consider the guides of specialized associations, such as family physicians or nursing. Although deciding what standard to use can be challenging to those designing and evaluating programs, evaluating without an understanding of the level of quality desired can lead to many complications and a waste of resources.

Communicating and reporting evaluation results are crucial to attaining the evaluation objectives [27, 68, 75]. Moreover, effective communication strategies have many important functions, such as providing decision makers with the necessary data to make an informed decision. Informing other stakeholders about the results is also important to achieve their support in implementing program changes and nourish a culture of quality [77, 78]. Around half of the authors of studies included in this review indicated that they were publishing to share their own evaluation experiences, while the other half did not. Regardless, none of the studies shared or indicated how their results were reported and communicated, which is an important part of the evaluation cycle that should not be overlooked when sharing evaluation lessons within the scientific community. Reporting the results also ensures quality transformation by closing the evaluation cycle and encourages future engagement in evaluation among different stakeholders [78–80]. Moreover, the results of the evaluation should be shared publicly to contribute to increasing public trust in educational programs and their outcomes [19, 69].

In summary, this review of evaluation practices within competency-based curricula for undergraduate and postgraduate health professional programs provides valuable insight into the current landscape. The results of the review show that most evaluation practices published pertain to postgraduate medical programs. In addition, by examining the objectives, models, tools, standards, and communication of evaluation results, this study exposes a discrepancy between the reported evaluation practices and identified evaluation elements. This discrepancy extends to the data that are reported, which makes it even more difficult to synthesize a holistic picture and definitively fulfill the aim of the review. Moreover, the issue of missing information poses serious challenges for educators who try to leverage existing knowledge to inform their curriculum development and improvement efforts, and it highlights the need for a more systematic and transparent approach to evaluation within CBME.

## Conclusion

This review illustrates the importance of agreeing on the main evaluation elements to be reported when publishing a CBME evaluation. Establishing a shared understanding of these fundamental elements will give educators a framework for enhancing the practical utility of evaluation methodologies. In addition, educators and practitioners can ensure that the evaluation process yields more insightful outcomes and is better tailored to meet the needs of the educational context.

### Electronic supplementary material

Below is the link to the electronic supplementary material.

Supplementary Material 1

Supplementary Material 2

Supplementary Material 3

## Data Availability

The datasets used during the current study are available from the corresponding author on reasonable request.

## Abbreviations

OBME: Outcome-Based Medical Education
CBME: Competency-Based Medical Education
EEM: Embedded Evaluation Model
