# Supplementary material for: Evaluating competency-based medical education: a systematized review of current practices
Source: BMC Med Educ. 2024 Jun 3;24:612. doi: 10.1186/s12909-024-05609-6 (PMC11149276; doi:10.1186/s12909-024-05609-6)
Supplement: Supplementary file 1 — Supplementary Material 1 [file 12909_2024_5609_MOESM1_ESM.docx]

Additional File 1. Query Terms Used by Librarian to Articles for Current Review

PUBMED

| **Resulted Query:** | **Filters Applied** | **Final Results** |
| --- | --- | --- |
| (Competency-Based Education[MeSH Terms]) AND (Program Evaluation[MeSH Terms]) AND (education, professional[MeSH Terms])  Search Details:  competency based education"[MeSH Terms] AND "program evaluation"[MeSH Terms] AND "education, professional"[MeSH Terms] | Full-text | 376 |

CINHAL

| **Resulted Query:** | **Filters Applied** | **Final Results** |
| --- | --- | --- |
| AB ( competency-based medical education or cbme ) AND AB education, medical AND AB ( evaluation or assessment or effectiveness or impact ) | Full-text; published after 2000 | 185 |

EDUCATION SOURCE

| **Resulted Query:** | **Filters Applied** | **Final Results** |
| --- | --- | --- |
| AB ( competency-based medical education or cbme ) AND AB education, medical AND AB ( evaluation or assessment or effectiveness or impact ) | Full-text | 65 |

ERIC

| **Resulted Query:** | **Filters Applied** | **Final Results** |
| --- | --- | --- |
| TX ( competency-based medical education or cbme ) AND TX education, medical AND TX ( evaluation or assessment or effectiveness or impact ) | Lang: eng - Peer Reviewed | 14 |
