# Supplementary material for: Evaluating competency-based medical education: a systematized review of current practices
Source: BMC Med Educ. 2024 Jun 3;24:612. doi: 10.1186/s12909-024-05609-6 (PMC11149276; doi:10.1186/s12909-024-05609-6)
Supplement: Supplementary file 2 — Supplementary Material 2 [file 12909_2024_5609_MOESM2_ESM.docx]

| **Additional File 1. Evaluating Competency Based Medical Evaluation: A Systematized Review of Current Practices**  **Data extraction tool that was designed and used to extract data from the articles included in the current systematized review.** | | | | | | | | | | | |
| --- | --- | --- | --- | --- | --- | --- | --- | --- | --- | --- | --- |
| **Author & Year** | **Institution** | **Country** | **Study aim** | **Study method** | **Type of CBME Curriculum / Program** | **CBME Implementation Level** | **Evaluation Objective / Question** | **Evaluation Approach (Model) / Tools** | **Evaluation Standard Used** | **Results of Evaluation** | **Evaluation Report / Sharing Results** |
|  |  |  |  |  |  |  |  |  |  |  |  |
|  |  |  |  |  |  |  |  |  |  |  |  |
|  |  |  |  |  |  |  |  |  |  |  |  |
|  |  |  |  |  |  |  |  |  |  |  |  |
|  |  |  |  |  |  |  |  |  |  |  |  |
|  |  |  |  |  |  |  |  |  |  |  |  |
|  |  |  |  |  |  |  |  |  |  |  |  |
