# Supplementary material for: Evaluating competency-based medical education: a systematized review of current practices
Source: BMC Med Educ. 2024 Jun 3;24:612. doi: 10.1186/s12909-024-05609-6 (PMC11149276; doi:10.1186/s12909-024-05609-6)
Supplement: Supplementary file 3 — Supplementary Material 3 [file 12909_2024_5609_MOESM3_ESM.docx]

| **Additional File 3. Overview of the characteristics and details of the data extracted from the studies included addressing evaluation practices in health professions education** | | | | | | | | | | | |
| --- | --- | --- | --- | --- | --- | --- | --- | --- | --- | --- | --- |
| **Author & Year**  **Study Title** | **Institution** | **Country** | **Article / Study aim** | **Study method** | **Type of CBME Curriculum / Program** | **CBME Implementation Level** | **Evaluation Objective / Question** | **Evaluation Approach (Model) / Tools** | **Evaluation Standard Used** | **Results of Evaluation** | **Evaluation Report / Sharing Results** |
| 1. Acai et al., 2020 | McMaster University | Canada | This study examined CC implementation at a Canadian institution, documenting the shared and unique challenges that CCs faced and overcame over a 3-year period, as well as adaptations and potential solutions with broader applicability. | This study consisted of three phases, which were conceptually and analytically linked using Moran-Ellis and colleagues’ notion of ‘following a thread.’ | Medical Residency Program  57 specialty and sub-specialty program | Postgraduate level | Not specified | Approach: Not Specified  A multi-method approach involving interviews, surveys and observations was used to examine CC implementation throughout the study period. | Not specified | The findings of this study reinforce the importance of resident engagement and information sharing between disciplines | Only publishing this article was reported. |
| 1. Battat et al., 2016 | The Haiti Medical Education (HME) Project, a non-profit organizatio | The Republic of Haiti | We present the creation, delivery and evaluation of a competency-based continuing medical education curriculum for physicians in rural Haiti. | The resulting educational goals were reviewed by a committee of Haitian and North American physician/medical education practitioners to reflect local needs. All authors reviewed lectures and then conferred to establish agreement on competencies presented for each lecture. | Medicine | Postgraduate level (Continuous Medical Education) | Not specified | Approach: Not specified  To assess the coverage of competency-based learning objectives, each lecture was analyzed individually and separately by two reviewers, who were blinded to the other’s evaluation | American Academy of Family Physicians (AAFP) and College of Family Physicians Canada (CFPC) guideline | We identified teaching goals covered and competencies that were missing from a CME program for rural Haitian physicians. We aim to use this analysis to provide a competency-based CME lecture series that proportionally meets local needs while following recommendations of recognized national family medicine organizations | Only publishing this article was reported |
| 1. Byung hwa lee et al., 2010 | International Cyber University for Health (ICUH) | Korea | Evaluate the effectiveness of a competency-based learning (CBL) approach to an e-learning course on systems analysis and design (SAD) | The competency of 18 students who registered for an SAD course was measured at different 3 times during the semester with the use of a competency diary.The changes in the competency scores through the semester were analyzed by a Friedman test, and the factors affecting learning effectiveness were identified by multiple regression. | Medicine Program | Undergraduate level | Aassess how well SAD competencies could be taught via a CBL-based e-learning course that we developed for students registered at the ICUH | Approach not specified  Tool: Course evaluation survey | Not specified | The competency scores increased as the semester progressed. The factors that had a significant effect on learning effectiveness were course management and learning materials.The authors found that the CBL approach worked well for this particular e-learning course on SAD and that nontechnical aspects of the instruction, such as course management and lecture materials, were more important than the technical aspects even in this Internet environment. | Only publishing this article was reported |
| 1. Cox et al., 2012 | C Change Organization awarded four universities:  Iowa Cancer Coalition (ICC), University of Florida (UF), Virginia Commonwealth University (VCU), and South Puget Inter- tribal Planning Agency (SPIPA) | USA | The results from the four grant sites and tools used to achieve them are described in this article. | Not specified | Medicine Program  Four diverse grant palliative care programs to yield gains in the management of pain and palliative care. | Postgraduate level | Not specified | C-Change developed competency standards and logic model-driven implementation tools | Convened by C-Change, a national, multi-disciplinary panel of leaders and experts developed competency standards that define the knowledge and skills needed by all healthcare providers. | The program participants achieved quantitative and qualitative improve- ments in their knowledge, skills, and attitudes. Similarly, the host organizations benefited from the experience, and in each instance developed tailored strategies to build on the accomplishments of the pilot projects. All sites reported that the competency tools demonstrated both flexibility and util- ity to meet the goals of their individual organizations. A full description of the standards, tools, and pilot site results can be found at www.cancercorecompetency.org. | Not specified |
| 1. Crawford et al., 2019 | Queen's University | Canada | Describe initial experiences in implementing CBME  to highlight perceptions and barriers and facilitate implementation at other centers  Research Questions  1. What are Queen's residents', Queen's programme leaders, and Canadian neurology programme directors' perceptions of CBE?  2. What are the current barriers to implementing CBE from the per-  spective of Queen's residents', Queen's programme leaders, and Canadian neurology programme directors? | Anonymous online surveys were administered to faculty and residents transitioning to CBE | Medicine:  Residency programs (Many specialties and sub-specialties) | Postgraduate medical education programs | Report on the early perceptions of CBE and the barriers experienced by faculty and residents both at Queen's and within Neurology  programmes across Canada | Approach Not specified  Tool: Surveys. | Not specified | Overall perceptions of CBE were favourable.  29.5% of participants were neutral or had little concern or no concerns about CBE implementation while 70.5% of participants were concerned with at least one or more barriers  Canadian neurology programme direc- tors (100%) and Queen's programme leaders (45.8%) indicated that “Access to adequate information technology” was a significant barrier to CBE implementation.  66.7% of Queen's programme leaders felt that “Failure of residents to take on a leadership role in CBE” was their greatest concern.  The greatest barriers perceived by Queen's residents were “Responsiveness of faculty to feedback” (39.2%) and “knowledge of which assessment to select” (31.1%). | Only publishing this article was reported. |
| 1. D’souza et al., 2014 | Western University | Canada | Determine the efficacy and effectiveness of incorporating an in- tegrated, kinesthetic, multidisciplinary team (MDT)-based head-and-neck educational program for radiation oncol- ogy post-graduate trainees, and to assess the effects of the educational program on competency development at multiple levels. | Prospective cohort research design | Medicine Program.  London Regional Cancer Program; Head-and-neck gross and radiologic anatomy program | Postgraduate Program | Assess the effects of the educational program on competency development at multiple levels. | Approach not specified  Tool: Pre – Post Questionnaire  Pre-post knowledge tests. | Not specified | Short-term core medical knowledge improvements with minimal procedural improvement in target delineation skills. Overall, results indicated positive satisfaction amongst participants. | Not specified |
| 1. Day et al., 2012 | National Pediatric Oncology Unit | Guatemala | This study measured immediate and short- term outcomes and constitutes the initial step in the process of comprehensive program evaluation | Not clearly specified | Nursing Program – Focus: Pediatric Oncology | Postgraduate level | Five outcome measures were assessed: (1) the rate of completion of a pediatric oncology nursing education course by newly hired nurses, (2) the rate of chemotherapy competency, (3) the rate of central-venous line care competency, (4) the hours of continuing education com- pleted, and (5) the cost in comparison to three other education models used in low-income countries. | Not specified | Not specified | A nursing quality assessment found no documentation of theoretical or clinical training for nurses within the Guatemalan National Pediatric Oncology Unit. Numerous factors contributed to this deficiency. There was no organized approach to education in the pediatric oncology unit, and nurses were not provided time or support for educational activities. The unit’s head nurse was responsible for educating newly hired nurses, but her many other responsibilities left little time to do so. Further, specialized training for nurses was not perceived as a high priority by the institutional leadership, and as a result financial support was unavailable. | Not specified |
| 1. De Beer, 2019 | The Royal Australian and New Zealand College | New Zealand | Identify those aspects of the RANZCP CPD programme that demonstrate compe- tency-based educational principles based on the four RCPSC features for a competency-based CPD programme, and those areas in which this approach is lacking. | Not applicable | Medicine:  Psychiatry | Continuing professional development (CPD) programme. | Not specified | Not specified | Royal Australian and New Zealand College of Psychiatrists Features of competency based CPD program. | The RANZCP have implemented a competency-based CPD programme that aligns with at least three of the components proposed by the RCPSC. The adoption of a competency framework (i.e. CanMEDS), a ‘curriculum’ of outcome-based learning objectives (defined by individual learners’ needs) and a ‘smorgasbord’ of various learning methodologies (to achieve learning outcomes) represent the three areas of CBEP implementation. | Not specified |
| 1. Ekenze et al., 2010 | West African College of Surgeons | West Africa | The aim of the study was to determine if pediatric surgery residency training program in West Africa addresses the realities of posttraining practice. | The study used a cross-sectional survey of 36 pediatric surgeons trained in West Africa using self-administered questionnaires | Medicine –  Pediatric Surgery | Postgraduate level | To evaluate the use of the surgical and nonsurgical areas of the training program, practicing fellows | Approach Not: specified.  Tool: Self-administered questionnaire | Not specified | Overall, 26 (72%) responded. Although 21 (81%) had adequate exposures in most surgical components of training, 18 (69%) were exposed to most of the nonsurgical components. The least in exposure and use were prenatal management, microvascular and laparoscopic surgeries, hospital administration, and finance management. Pediatric urology, gastroenterology, oncology, trauma, neonatal surgery, burn management, and hepatobiliary surgery were rated as useful and relevant to practice by 22 (85%) of the respondents. Many nonsurgical areas that receive less emphasis in training were regarded as useful in practice by all respondents including ethical decision making, accessing scientific literature, communication skills with colleagues and patient's guardian, and medical research. Significant challenges to training were lack of tertiary children's hospital, dearth of facilities, and inadequate mentoring | Not specified |
| 1. Ellaway et al., 2018 | Several Medical Schools (16 of Canada’s 17 medical schools) | Canada | Triple C is the Canadian competency- based medical education (CBME) initiative for family medicine. The authors report on a study exploring the impacts Triple C has had across Canada. | Interviews and focus groups with key medical school stakeholders. Data were analyzed using thematic and template analysis techniques. | Medicine –  Family medicine | Postgraduate level | Explore the impact of Triple C (Canadian CBME imitative for family physician) implementation in different programs across Canada. | Pawson’s model of realist program evaluation  Tool: Interviews and focus groups. | CanMEDS-FM framework | We found that, as of 2016, Triple C implementation was a patchwork of different interpretations and compromises that reflected different local enablers and inhibitors in the many different contexts of Canadian FM training. As a result, the impact of Triple C varied both between programs and within a single program (according to the training site involved). | Not specified |
| 1. Fahim et al., 2016 | McMaster University | Canada | The primary focus of this study was to develop and pilot an evaluation tool that provides effective, formative feedback to assess each of the CanMEDS competencies, in the context of grand rounds. | This study employed the use of a 3-phase,  Qualitatively focused, embedded mixed methods approach. | Medicine:  Surgery Residency Program | Postgraduate level | Not specified | The proposed evaluation tool contains 13 Likert scale questions and 2 open-ended questions. The tool  outlines specific questions to assess grand rounds presenters.  within the structure of the 7 CanMEDS competency  domains. | CanMEDs | This study demonstrates that the CanMEDS framework can be effectively integrated into the evaluation of academic presentations, and adapted to include the assessment of senior sessions, journal clubs, and chair rounds. | Only publishing this article was reported. |
| 1. Freedman et al., 2014 | The Emory Public Health Training Center (PHTC) | USA | This article describes how the Emory PHTC uses its logic model to guide development of a comprehensive evaluation plan and to create specific data collection tools. | Not applicable | Public Health Program | Unclear | assess what is working, what is not working, and why; to guide decision making for continuous quality improvement; and to ensure efficient use of resources to strengthen the public health workforce. | The plan, based on the center’s logic models, includes formative assessment, outcome evaluation, process evaluation, and overall programmatic evaluation.  Typical data collection tools include survey instruments, inter- view scripts, and focus group guides. | Not specified | Evaluation data have informed improvements in instructional design, choices of faculty instructors, course curricula, and training locations—all of which have supported the center’s overarching goal of strengthening the current and future public health workforces. | Only publishing this article was reported |
| 1. Gibson et al., 2008 | University of New South Wales | Australia | To design a comprehensive, multicomponent, program-wide evaluation and improvement system. | Not applicable | Medicine:  Outcome Based Medical Program | Undergraduate level | (1) evaluate the effectiveness of the change, (2) monitor its implementation to enable continual improvement, and (3) use evaluation to recognize and report on excellence in teaching. | Self-formulated six strategic principles to guide development of a program evaluation and improvement strategy  Tool: Survey | Accreditation Standards of the Australian Medical Council | The creation of an approach that evaluates the quality of the program in terms of four main and related aspects: curriculum and resources, staff and teaching, student experience, and student and graduate outcomes. Twenty-three key quality indicators aligned with these four aspects were identified and provide a broad, but manageable framework to guide the development and implementation of sustainable program evaluation and improvement processes. | Only publishing this article was reported. |
| 1. Goudreau et al., 2009 | University of Montreal | Canada | The purpose of this article is to report on a curriculum innovation. A description of the background of the reform and a discussion of the fundamentals of the second-generation CBA will be followed by a presentation of how the approach is applied in the program, and more specifically, in one course in the program. | Although no rigorous evaluation has been held, comments from students and teachers were collected during the implementation process. Assessment of students’ competency levels remains an important issue. | Bachelor Program in Nursing | Undergraduate level | Not specified | Approach: Not specified  Course evaluation was conducted using a self-administered student questionnaire, student focus groups, and teacher focus groups. | Not specified | The evaluation data clearly indicate that the second- generation CBA contributes added value to the education of future nurses. | Not specified |
| 1. Gruber et al., 2007 | The Chinese University of Hong Kong | China | To describe a course designed to help medical undergraduates develop the necessary competencies to recognise and manage acutely ill patients. | Primary description by the authors of the content, development and implementation of a course designed to teach competencies recommended by the Acute Care Undergraduate Teaching (ACUTE) project of the Resuscitation Council (UK) and Intercollegiate Board of Training in Intensive Care Medicine. | Medical Program.  Acute care project | Undergraduate level | Not specified | Approach: Not specified  Tool: Students were asked to rate anonymously aspects of the course on a scale of 1—5 where 5 indicated excellence. | Not specified | The course addresses 55/71 (77%) of competencies considered important by the ACUTE project. It was well accepted by medical students and on a scale of 1 (poor) to 5 (excellent) median student ratings of various components of the course ranged from 4—5. | Not specified |
| 1. Hamza et al., 2019 | College of Family Physicians of Canada | Canada | Present a case for the following: (a) the development of a program theory is essential prior to or in the initial stages of implementation of CBME; (b) the program theory should guide the strategies and methods for evaluation that will answer questions about anticipated and unintended outcomes; and (c) the iterative process of testing assumptions and hypotheses will lead to modifications to the program theory to inform best practices of implementing CBME. | Using a mixed methods design, the processes and outcomes of Triple C were explored through surveys, interviews, and historical document review, which captured the experiences of various stakeholders. | Medicine Program – Focus on family medicine residency | Postgraduate level | The development of the program theory of Triple C initially focused on identifying how family medicine residency education should be improved by using a social accountability approach to: “address the priority health concerns of the community, region, and/or nation (Canada). | Theory-based evaluation approaches  Tools: Mixed method (surveys, interviews, and historical document review,) | Not specified | The theory-led program evaluation process was able to identify areas that supported CBME implementation: the value of a strong nondirective national vertical core supporting the transformation in education, program autonomy, and adaptability to pre-existing local context. Areas in need of improvement included the need for ongoing support from College of Family Physicians of Canada (CFPC) and better planning for shifts in program leadership over time. | Only publishing this article was reported |
| 1. Ilhan, 2021 | Coded | Turkey | Evaluate competency based medical education curriculum. | Qualitative study is designed as a case study. | Medicine Program | Undergraduate level | 1. What are the views of the faculty members and the students about the CBME curriculum?  2. What are the views of the faculty members and the students about the implications of the CBME curriculum?  3. What are the opinions of the faculty members and the students about the effects of CBME in terms of academic/vocational developments of students? | Stufflebeam’s CIPP model.  The study group consisted of fifteen faculty members and fifty students of a medical school in Turkey. The data was collected via semi-structured interview forms and analysed applying content analysis. | Not specified | The students have had mostly negative ideas about the competencies and the teaching-learning process while the faculty members have been satisfied with their practices. Both the students and the faculty members thought the CBME contributed to students’ academic and vocational developments | Only publishing this article was reported |
| 1. Janssen et al., 2005 | B.C. Women’s, a tertiary-level teaching maternity hospita | Canada | To evaluate the success of a competency-based nursing orientation programme | A prospective cohort design comparing self-reported competencies prior to starting work in the single-room maternity care and six months after. | Nursing Program – Maternity Care | Postgraduate Level | Measuring improvement in self-reported competency after six months. | Approach: Not specified  Tool: The Perinatal Nursing Self-Efficacy Survey  In addition, a new tool was designed, the Single-room maternity care (SRMC) Competency Tool | Not specified | Scores on the perinatal self-efficacy scale and the single-room maternity care competency tool were improved. These differences were statistically significant. | Only publishing this article was reported |
| 1. Kerfoot et al., 2004 | Massachusetts General Hospital, Brigham and Women’s Hospital, and Beth Israel Deaconess Medical Cen- ter | USA | Evaluated urological learning by medical students using a validated measure of  learning in 4 clinical areas. | Pre and post assessment and evaluation surverys | Medicine Program  Focus on Urology | Undergraduate level | Not specified | Approach: Not specified  Tool: Pre and post assessment and evaluation surverys | Not specified | This study demonstrates significant learning by medical students during their 1-week clinical rotation in urology. | Not specified |
| 1. Ketteler et al., 2014 | The University of New Mexico | USA | Our aim was to create a CCC that would meet the following 4 criteria: (1) be centered on the milestones, (2) be simple and efficient in assessment milestone achieve- ment for semiannual resident review, (3) establish faculty expertise in each competency (“competency champions”), and (4) help coach and remediate residents who are not progressing appropriately based on their milestone perform- ance evaluations. | We created a CCC that meets monthly and at each meeting reviews a resident class for milestone perform- ance, a competency (by a faculty competency champion), a resident rotation service, and any other resident or issue of concern. | Medicine: General Surgery Residency Program | Postgraduate level | Not specified | Not specified | ACGME Competencies and the American Board of Surgery | The function of our CCC has also allowed us opport- unity to evaluate the required rotations to ensure that they offer experiences that help residents achieve competency performance necessary to be safe and effective surgeons upon completion of training. | Not specified |
| 1. Koeijers et al., 2012 | Saint Elisabeth Hospital | Dutch Caribbean islands | Evaluate whether a competency-based curriculum implemented in a Caribbean teaching  hospital fulfilled the requirements as defined by the CanMEDS framework. | A triangulation method in the survey to obtain information on the educational process. | Medicine Program - | Undergraduate level | Evaluate whether a competency-based curriculum implemented in a Caribbean teaching.  hospital fulfilled the requirements as defined by the CanMEDS framework. | Approach: Not specified  Tools: surveys  Site visits / assessment of educational activities by two reviewers. | Not specified | Major recommendations included increased involvement of medical specialists in the educational activities in the clinical workplace.  There was need for improvement of communication between medical specialists, patients, nurses, trainees and residents. Overall, improvements were observed in the structure of clinical rotations and content of the training programme. | Only publishing this article was reported |
| 1. Lipp, 2008 | The New York University College of Dentistry | USA | This article describes the course and principles fundamental to its design and presents data based on student performance measures and surveys/course evaluations | Not applicable | Dentistry  Focus on malocclusion and skeletal problems | Undergraduate level | Evaluate malocclusion and skeletal problems. | Approach: Not specified.  Tool: Students course evaluation - Post-course meetings | Not specified | Not specified | Only publishing this article was reported |
| 1. Nousiainen et al., 2018 | University of Toronto | Canada | Describe the journey toward CBME, the challenges faced, the benefits enjoyed, and the results observed after eight years of operating within the new framework. | Not applicable | Medicine –  Orthopaedic Surgery Residency Program | Postgraduate level | Not specified | Approach: Not specified  Tools : Curriculum mapping, feedback from external reviews from accrediting bodies. | Accreditation body in the competency-based curriculum | Feedback from the residents, the faculty, and the postgraduate residency training accreditation bodies on the CBC has been positive and suggests that the essential framework of the program may provide a valuable tool to other programs that are contemplating embarking on transition to competency-based education. | Not specified |
| 1. Railer et al., 2020 | Queen's University | Canada | To assess the efficacy of CBME implementation through a programme evaluation process, , which included the use of outcome harvesting (Wilson-Grau) | Outcome harvesting; A systematic approach to document analysis was used to categorize the eight identified areas of implementation: governance, scholarship, faculty development, resident leadership, curriculum, assessment, communications, and technology. | 29 Residency programs – Royal college of Physicians and Surgeons of Canada Specialty Programs | Postgraduate medical education programs | Evaluate efficacy of CBME implementation and uncover the outcomes of the implementation of a CBME curriculum:  Q1. In which way did social actors involved with CBME influence the change to CBME curriculum?  Q2. How do the outcomes represent progress towards the shift to a CBME curriculum?  Q3. What were the intended and untended consequences of implementing CBME in postgraduate medical education at Queen's? | Approach: Concerns-Based Adoption Model, Sensemaking, and Outcome harvesting.  Tool:  Document analysis. | Not specified | In response to the evaluation questions, the study harvested 38 outcomes from the document analysis. | Only publishing this article was reported. |
| 1. Randolph et al., 2011 | Coded | USA | Th is article addresses competency achievement o f M P H and MS graduates who were enrolled both on-campus and through distance education. | A descriptive multi-point cross-sectional study | Nursing –  Occupational health nursing program | Postgraduate level | Evaluate self-reported competency achievement by occupational health nursing program graduates. | Twelve competencies were evaluated at three levels: compe-tent, proficient, and expert.  A survey tool to measure competency achievement of occupational health nursing program graduates | The American Association of Occupational Health Nurses, Inc. "Competencies in Occupational and Environmental Health Nursing," scope of practice | Distance education learners had higher competency scores compared to on-campus graduates.  Reported competency achievement increased in all areas except research.  Based on competency findings. curriculum and course assignments related to leadership role, policy development, professional devel- opment, and research were modified. | Not specified |
| 1. Rotthoff et al., 2012 | Heinrich-Heine University | Düsseldorf, Germany | The aim of the present study was the development and psychometric evaluation of an MS-Questionnaire (MSQ) focusing on explicit competencies | A questionnaire was derived from the MS “teaching” (Medical Faculty, Heinrich-Heine University Düsseldorf) which was based on (inter-) nationally accepted goals and recommendations for a competency based medical education.  The MSQ was administered together with the Dundee Ready Education Environment Measure (DREEM) | Medical program | Undergraduate level | To what extent the MSQ captures the construct of learning environment and how well a faculty is following in its perception a competeny orientation in a competency based curriculum. | Approach: Not specified  Tool: Questionnaire.  the Dundee Ready Education Environment Measure (DREEM) | Not Specified | Students and teachers perceived the MS implementation as “moderate” and on average, students differed significantly in their perception of the MS. They thought implementation of the MS was less successful than faculty did. Women had a more positive perception of educational climate than their male colleagues and clinical students perceived the implementation of the MS on all dimensions significantly worse than preclinical students | Only publishing this article was reported |
| 1. Schonwetter et al., 2011 | University of Manitoba | Canada | The is the first initiative of a larger project that focused on the assessment of dental program by various stakeholders, including new students, current students, graduates, alumni, and the dentist community. In addition, contribution of each of the three learning environments (classroom, clinic, and externship) towards competency development was assessed. | A questionnaire examining graduating dental students’ confidence and perceived importance of 47 competen- cies expected by the ACFD/CDA by requiring students to rate each competency on a five-point Likert scale | Dental Program | Undergraduate Program | Not specified | Approach not Specified  Tool: Questionnaire | Association of Canadian Faculties of Dentistry (ACFD) and the Commission on Dental Accreditation of Canada (CDAC) | Strong affirmation of the importance of the current CDAC global competencies for beginning dental practitioners. The programme demon- strates high perceptions within the students that the current dental programmes over a period of 5 years is eliciting the significance of the competencies as important for dentistry. | Not specified |
| 1. Stefanidis et al., 2008 | Department of General Surgery, Carolinas Medical Center | USA | The purpose of this article is to describe our experience with the incorporation of a proficiency-based laparoscopic skills curric- ulum in a busy surgical training program that aims to improve the technical proficiency of residents. | Not applicable | Medicine Program - Laparoscopic Surgery program  Focus: Laparoscopic Surgery program | Postgraduate level | To use the following outcome measures to monitor the effectiveness and the efficiency of the curriculum include :resident attendance rates, performance improvement at the end of the academic year compared with baseline, and cost data | Approach: Not specified  Tool: Participant performance is evaluated with objective metrics. Task duration and errors comprise the metrics for the 5 Fundamentals of Laparoscopic Surgery tasks. or the 9 virtual reality tasks, the task duration, errors, and motion efficiency metrics are used for evaluation | Not specified | The attendance rates increased dramati- cally after the incorporation of these measures, from 6% to 71%. In regard to resident performance, residents have found the combination of standard and best training goals very chal- lenging and motivating so far. Some have already beaten the best scores on some tasks. | Not specified |
| 1. Stucke et al., 2018 | Two academic institutions: Dartmouth Hitch- cock Medical Center (DHMC) and University of Wisconsin-Madison (UW) | USA | There is currently no evidence-based definition of competence for the consultation and no existing framework for the evaluation of the surgical consult. Our group sought to define competence for sur- gical consultation, and to determine how the consult is currently evaluated. | Qualitative interviews performed with 23 surgical faculty at two academic institutions. In- terviews were reviewed for thematic content. | Medicine – Surgery | Postgraduate level | Not specified | Approach: Not specified  Tools: Interviews | Not specified | No explicit framework is currently used to evaluate the surgical consult. Most participants currently use subjective, global performance assessment. This method often relies on information not limited to the discrete consult at hand. Competence for a discrete surgical consult can be defined by six key procedural steps and six performance traits. Five red-flag behaviors were identified that negatively impact entrustability. | Not specified |
| 1. Succar et al., 2017 | The university of Sydney | Australia | To evaluate innovative educational strategies that help op- timize ophthalmology teaching in a crowded medical curriculum. | A mixed-methods research design was employed to include both quantitative and qualitative dimensions in evaluating the revised curriculum with medical students (n = 328) undergoing their ophthalmol- ogy rotation. | Medicine Program – Ophthalmology rotation. | Undergraduate level | Exploring and analyz- ing medical students’ academic performance and education- al experience in the revised ophthalmology curriculum | Approach: Not specified  Tool: Quantitative evaluation was performed with a 20-item mul- tiple choice pre- and post-test of ophthalmic knowledge. a 12-month fol- low-up test was readministered to compare the long-term retention rate of graduates. Qualitative evaluation was measured with student satisfaction questionnaires. | The association of university Professors of Ophthalmology MedEd task Force has also approved core knowledge and skills competencies, and the royal College of Ophthalmologists provides guidance on competencies for under- graduate ophthalmology teaching. | In the original curriculum there was an improvement of 19.9% from pre- to post-test scores and a greater improvement of 31.6% from pre- to post-test in the revised curriculum.  When assessing retained knowledge it increased in the revised curriculum.  In addition, qualitative feedback also improved, with the rota- tion being highly valued. | Not specified |
| 1. Swider et al., 2006 | Rush University | USA | This paper describes a process for evaluation and revision of a graduate curriculum to prepare Advanced Practice Clinical Nurse Specialists (CNS) in P/CHN, to ensure that the educational program addresses and develops knowledge and proficiency in all relevant competencies. | Quantitative and qualitative data are collected using multiple methods and sources, which include students, graduates/alumni, faculty, preceptors, Advisory Committee members, and em- ployers. | Nursing Program  Advanced Practice Clinical Nurse Specialists (CNS) in P/CHN | Postgraduate  Program | Ensure that the educational program addresses and develops knowledge and proficiency in all relevant competencies. | Approach: CIPP (context, inputs, proc- esses, and products) model  Tool:  Quantitative and qualitative data are collected using multiple methods and sources, which include students, graduates/alumni, faculty, preceptors, Advisory Committee members, and em- ployers | Quad Council Competencies  Competencies of the American Association of Colleges of Nursing Master’s Essentials, the National Organization of Nurse Practitioner Faculties Core | The faculty believe that the evaluation process was of overall benefit to the program. The benefits include the fact that the resulting curriculum has enhanced relevance to clinical practice. In addition, the evaluation process forced faculty to gain expertise in the competencies, and to consider the competen- cies in relation to teaching/learning principles. | Not specified |
| 1. Taleghani et al., 2004 | Baylor College of Dentistry | USA | The objective of this article is to report the development, implementation, and early results of a non-graded normative dental student clinical performance assessment system based on our competencies documents. | Every step of each clinical procedure was recorded, weekly performance summaries by both students and faculty were collected, and periodic workshops were held to refine the forms and further calibrate faculty. | Dental Program | Undergraduate level | Pinpoint and document performance deficiencies that could lead to clinical failure. | Tool: Faculty and student evaluation surveys | Not specified | we believe the new system has provided a better environment for students and fac- ulty alike and will be a very positive factor in sup- port of Baylor’s “Lifelong Colleague” initiative for the practicing community. | Only publishing this article was reported |
| 1. Thoma et al, 2020 | Not specified | Canada | Evaluate Canadian postgraduate specialist Emergency Medicine (EM) programs short-term educational outcomes nationally and within individual programs. | Program-level data from the 2018 resident cohort were amalgamated and analyzed. The number of Entrustable Professional Activity (EPA) assessments (overall and for each EPA) and the timing of resident promotion through program stages was compared between programs and to the guidelines provided by the national EM specialty committee. Total EPA observations from each program were corre- lated with the number of EM and pediatric EM rotations. | Medicine – Emergency Medicine | Postgraduate | Not specified | Approach: Not specified  Tools:  Program-level data from the 2018 resident cohort were amalgamated and analyzed. | Not specified | We present a new approach to the amalgamation of national and program- level assessment data. There was demonstrable variation in both EPA- based assessment numbers and promotion timelines between pro- grams and with national guidelines. This evaluation data will inform the revision of local programs and national guidelines and serve as a starting point for further reaching outcome evaluation. This process could be replicated by other national assessment programs. | Only publishing this article was reported |
| 1. Van Melle et al., 2019 | Queen’s University | Canada | Examining “fidelity of implementation”—that is, whether CBME is being implemented  as intended—is hampered, however, by the lack of a common framework. This article details the development of such a framework. | A two-step methods was used. First, a perspective indicating how CBME is intended to bring about change was described. Accordingly, core components were identified. Drawing from the literature, the core components were organized into a draft framework. Using a modified Delphi approach, the second step examined consensus amongst an international group of experts in CBME. | Not specified | Not specified | Has CBME been implemented as intended ? | Approach: Not specified  Tools: Not specified | Not specified | Not applicable | Not specified |
| 1. Van Zuilen et al., 2008 | University of Miami Miller School of Medicine | USA | Describes the de- sign, development, implementation, and evaluation of this curriculum, reviews our data-driven curriculum quality improvement efforts, and discusses the challenges to translating student competency into routine practice. | The ADDIE instructional design model (Analysis, Design, Development, Implementation, Evaluation) and the continuous quality improvement (QI) model (Dew & McGowan Nearing, 2004) for program and process improvement | Medicine Program  The focus was on core areas of geriatrics. | Undergraduate level | Not specified | Quality improvement (QI) model (Dew & McGowan Nearing, 2004) for program and process improvement  Tools:  Likert-type satisfaction and usability surveys to evaluate the online and preceptor-led instructional and assessment activities.  Focus groups of faculty and students to answer questions about instructional content and delivery. | The Florida Consortium for Geriatric Medical Education (FCGME) | - Student and faculty evaluations reveal high levels of satisfaction with the curricular elements. - The QI efforts pro- vide and document continuing improvements in student competence in sequential cohorts of students and, in some cases, have allowed us to raise the performance standard for a small group of learning objectives. - Several faculty retreats were held after all curricular elements were implemented to analyze the ultimate effectiveness and efficiency of the curriculum. The following key issues and lessons were learned: - The recognition and initial evaluation and management of memory and mobility problems should remain a central focus of the curriculum. - Curriculum is overcrowded. - Not all learning objectives are core.Many of the learning objectives considered to be core by faculty fall within the FCGME domain of diagnosis and evaluation and require students to demonstrate a skill or apply knowledge - Medical Students’ Learning Is Part of a Continuum.   Competency May Not Translate into Habitual Practice | Only publishing this article was reported. |
| 1. Westein et al., 2019 | Not specified | Netherlands | The aim of this study was to describe the development, evaluation, and revision of the two-year postgraduate cur- riculum for community pharmacists in the Netherlands. | A two-year workplace-based curriculum was built. A development path along four milestones was constructed using 40 entrustable professional activities (EPAs). The assessment program consisted of 155 workplace-based assessments, with the supervisor serving as the main assessor. Also, 360-degree feedback and 22 days of classroom courses were included in the curriculum. In 2014, the curriculum was evaluated by two focus groups and a review committee. | Community Pharmacists Specialization Program | Postgraduate level | Based on input from surveys held earlier with trainees and supervisors, three themes were defined to evaluate in depth: trainees and supervisor workload, learning opportunities in the pharmacy workplace, and utility of the assessment system.  The participants were asked to identify bottlenecks they experienced and possible ways of improving the curriculum. | Evaluation Approach: Not specified.  Evaluation tool: Surveys then two focus groups and a review committee | Dutch Advisory Board for Postgraduate Curriculum Development for Medical Specialists were used as a guideline and Canadian Medical Education Directions for Specialists (CanMEDS) | Based on feedback from pharmacy supervisors, trainees, and other stakeholders, 22.5% of the EPAs were changed and the number of workplace-based assessments was reduced by 48.5%. | Only publishing this article was reported. |
| 1. Zhang et al., 2014 | Not specified | China | This article presents a successful teaching evaluation method for vocational CBE curricula since reforming teaching evaluation of medical linguistics courses for medical students by using the qualitative research method in education science. | Qualitative research method in education science is employed herein and the teaching results are evaluated according to students’ comments after attending the elective course, Medical Linguistic | Medicine program  Focus on medical linguistics | Undergraduate level | Evaluate teaching of the curricula for medical students’ vocational competency-based education (CBE) | Approach: Not specified  Tools: Qualitative research method / Further details not specified. | Not specified | Practice shows that Medical Linguistics is very popular with medical students and improvements have been made in educating them in essential vocational qualities. | Only publishing this article was reported. |
| 1. Zhang et al., 2019 | Not specified | Canada | A program evaluation plan was launched by the CFPC alongside the implementation of Triple C to explore if intended outcomes were achieved. | We conducted retrospective secondary data analysis of survey find- ings from graduating family medicine residents from two sources: National Physician Survey (NPS 2007 and 2010); and the Family Medicine Longitudi- nal Survey (FMLS 2015). Demographics and practice intentions reported by residents in the NPS 2007, NPS 2010, and FMLS 2015 were included in the analyses and a comparison between years was undertaken using a series of Pearson χ2 test. | Medicine Program – Focus on Family Medicine | Postgraduate level | The first is to provide results on outcomes from the implementation of a CBME curriculum by comparing responses from graduates of FM resi- dency programs across Canada pre and post-Triple C implementation, specifically in the area of their inten- tions to practice across a broad range of clinical domains. The second goal of this paper is to offer a case study of the feasibility of using historical data acting as a proxy for baseline data in comparing outcomes pre- and postimplementation of a CBME pro- gram in the absence of deliberately collected baseline data | Approach: Not specified  Tools:  Retrospective secondary data analysis / Surveys. | Not specified | Family medicine graduates report an increase in intention to include a broader range of clinical domains after implementation of Triple C. While a causal relationship cannot be determined, using a historical control in the form of survey data that predates Triple C implementation could support future approaches to evaluation of education reform. | Only publishing this article was reported. |
